# Poor performance on the Iowa gambling task in children with obsessive-compulsive disorder

**DOI:** 10.1186/1744-859X-11-25

**Published:** 2012-10-12

**Authors:** Masaki Kodaira, Yoshitaka Iwadare, Hirokage Ushijima, Arata Oiji, Motoichiro Kato, Nobuhiro Sugiyama, Daimei Sasayama, Masahide Usami, Kyota Watanabe, Kazuhiko Saito

**Affiliations:** 1 Department of Child and Adolescent Psychiatry, National Center for Global Health and Medicine, Kohnodai Hospital, 1-7-1 Kohnodai, Ichikawa, Chiba, 272-0836, Japan; 2 Department of Psychiatry, Kumamoto University Graduate School of Medicine, 1-1-1 Honjyo, Chuo-ku, Kumamoto City, Kumamoto, 860-8556, Japan; 3 Department of Developmental Psychiatry, Kitasato University Graduate School of Medical Science, 1-15-1 Kitasato, Minami-Ku, Sagamihara City, Kanagawa, 252-0374, Japan; 4 Department of Neuropsychiatry, Keio University School of Medicine, 35 Shinanomachi, Shinjuku-ku, Tokyo, 160-8582, Japan; 5 Department of Neuropsychiatry, Shinshu University School of Medicine, 3-1-1 Asahi, Matsumoto, Nagano, 390-8621, Japan

**Keywords:** Obsessive-compulsive disorder, Childhood-onset, Iowa gambling task, Wisconsin card sorting test, Orbitofrontal cortex

## Abstract

**Background:**

Several lines of evidence implicate orbitofrontal cortex dysfunction in the pathophysiology of obsessive-compulsive disorder (OCD). The purpose of this study was to investigate neuropsychological dysfunction of the orbitofrontal cortex in children with OCD.

**Methods:**

The Iowa Gambling Task (IGT), which reflects orbitofrontal cortex function, and the Wisconsin Card Sorting Test (WCST), which is associated with functioning of the dorsolateral prefrontal cortex, were administered to 22 children with OCD and 22 healthy controls matched for gender, age, and intelligence.

**Results:**

OCD patients displayed poor performance on the IGT. In contrast, performance on the WCST was not impaired in OCD patients compared to controls.

**Conclusions:**

These findings are in line with previous studies demonstrating that OCD in childhood is associated with a dysfunction of orbitofrontal-striatal-thalamic circuitry.

## Introduction

The current dominant model of obsessive-compulsive disorder (OCD) focuses on abnormalities in cortico-striatal circuitry, with particular emphasis on the orbitofrontal-striatal-thalamic circuit [1,2]. In particular, the orbitofrontal cortex (OFC), which facilitates behavioral flexibility upon negative feedback, plays a central role in most neurobiological models of OCD. For example, neuroimaging studies demonstrated that the activation of the lateral OFC along with several other cortical regions was abnormally reduced during a reversal learning task in OCD patients [3].

Performance on the Iowa Gambling Task (IGT), which evaluates real-life decision-making abilities under conditions of ambiguity, is particularly sensitive to alterations in OFC function [4,5]. In comparison, performance on the Wisconsin Card Sorting Test (WCST), which measures executive function or abstract reasoning abilities, is thought to be associated with functioning of the dorsolateral prefrontal cortex (DLPFC) [6], a region not included in the orbitofrontal-striatal-thalamic circuitry model of OCD [7]. Previous studies have revealed impaired IGT performance in adult OCD patients [8-11], which may reflect OFC dysfunction in these individuals. However, to our knowledge, no study has investigated IGT performance in children with OCD. Recent findings suggest that gray matter and white matter changes in children and adolescents with OCD are broadly consistent with those identified in adult OCD patients [12,13]. Therefore, we presumed that children with OCD might show impaired performance on the IGT, reflecting OFC dysfunction. Conversely, children with OCD may perform normally on the WCST if DLPFC function is not specifically associated with OCD in children.

In the present study, to test this hypothesis, we measured IGT and WCST performance in children with OCD and compared these results with those of gender-, age-, and intelligence-matched healthy, non-clinical controls. We excluded OCD patients comorbid with pervasive developmental disorder (PDD) or attention-deficit/hyperactivity disorder (ADHD) because a comorbidity of these disorders is known to affect performance on neuropsychological tests [14,15].

## Methods

### Subjects and clinical assessments

The study sample consisted of 22 Japanese OCD patients (between the age of 10 and 15 years). They were admitted as inpatients or outpatients at Kohnodai Hospital, National Center for Global Health and Medicine. Two trained child and adolescent psychiatrists made diagnoses following DSM-IV-TR [16] criteria. Participants with a current diagnosis of mood disorder, psychotic disorder, substance-related disorder, attention-deficit and disruptive behavior disorder, pervasive developmental disorder, or mental retardation were excluded from this study.

Normal healthy controls were recruited from 2 local public schools by word of mouth. A total of 47 students volunteered to participate, of which 2 were excluded due to obsessive symptoms, 1 due to tic disorder, and 3 due to pervasive developmental disorder. After matching for gender, age, handedness, and intelligence with the patient group, 22 were included in the analyses. Those with a current or past history of psychiatric diagnosis, psychiatric or psychological treatment, or absenteeism from school were excluded from the control group. A child psychiatrist conducted a 30-minute clinical interview with each control subject to rule out any current or past history of psychiatric disorders.

### Assessment

Two trained child and adolescent psychiatrists assessed the severity of OCD symptoms using the NIMH-Global Obsessive Compulsive Scale (NIMH-OCS) [17] in both OCD patients and controls, and also via the Children Yale-Brown Obsessive Compulsive Scale (CY-BOCS) [18] in the OCD group. The handedness of each subjects was determined using the Hand Usage Questionnaire [19].

The Iowa Gambling task (IGT) [20] was administered by one trained child and adolescent psychiatrist following the procedures of the original version. Previous studies have used the IGT to assess decision making in children and adolescents with ADHD [21], psychopathic tendencies [22], and self-harm tendencies [23]. One difference from the original task was that play money was converted from U.S. dollars to Japanese yen [24]. The IGT requires subjects to repeatedly select cards (100 in total) from four decks of cards and subjects are instructed to maximize their profit. The aim of the game is to win as much money as possible, or, as far as possible, to avoid losing money. To achieve this, subjects must discover which are the most advantageous decks and preferentially select cards from those decks. Each time they turn over a card, subjects will win some money; however, on turning over each card they also will sometimes have to pay a penalty according to a pre-programmed schedule of reward and punishment. Gains and losses are different for each card selected from the four decks. Decks A and B are “disadvantageous” because whilst they receive 10000 yen, the penalties are also higher, so they cost more in the long run (these decks are also termed high-paying decks). Decks C and D, on the other hand, are “advantageous” because whilst they receive only 5000 yen, the penalties are also lower, resulting in an overall gain in the long run (and are therefore termed low-paying decks). Thus, successful task performance relies on sampling more from decks C and D than from decks A and B. In the present study, subjects were informed that they could get a special gift if their money on hand exceeded 300,000 yen at the end of the task. The 100 selections were divided into five blocks of 20 selections each, allowing us to verify changes in the pattern of selections throughout the course of the experiment. We registered the number of disadvantageous cards selected in total, and also in each block of 20 selections.

Four clinical psychologists individually administered the Wechsler Intelligence Scale for Children-Third Edition (WISC-III) and the Keio version of the Wisconsin Card Sorting Test (WCST) [25]. WCST is a test for executive function or abstract reasoning involving working memory [6]. It is also a sensitive test of dorsolateral prefrontal cortex function [26]. In the WCST, categories achieved (CA), total errors (TE), and perseverative errors (PE) of Nelson were evaluated [27]. Categories achieved indicates the ability to change abstract categories, and perseverative errors represent the perseveration of preceding errors or the disturbance of inhibition of preceding incorrect responses.

### Statistical analyses

Data were analyzed using the Statistical Package for the Social Sciences (SPSS) Version 20 for Mac. T-tests were used to compare continuous variables (i.e., age and IQ), and Mann–Whitney tests were used to compare ordinal variables. The results were considered significant at p < 0.05.

### Ethical considerations

This study was designed in accordance with the Declaration of Helsinki and approved by the Ethics Committee of the National Center for Global Health and Medicine. All participants gave written informed assent and their parents gave written informed consent after the procedure had been explained to them.

## Results

Demographic and clinical characteristics are summarized in Table 1. The patients had been prescribed the following medications: sertraline (6 patients), paroxetine (2), and clomipramine (2). The following comorbidities were observed in the patients: tic disorders (3), trichotillomania (3), stuttering (1), and somatization disorder (1). No significant differences were observed between patients and controls in terms of age, gender, full scale IQ, or handedness. OCD patients scored significantly higher on the NIMH-OCS and lower on performance IQ compared to the controls.

**Table 1:** Subject characteristics and performances on the WCST

**Variable**	**OCD (n = 22)**	**Controls (n = 22)**	P value^*^
	**Mean ± SD**	**Mean ± SD**	
Gender			.62
Boy	12 (54.5%)	12 (54.5%)	
Girl	10 (45.5%)	10 (45.5%)	
Age, months	163.5 ± 22.1	161.8 ± 20.6	.27
Illness duration,	23.9 ± 21.3		
months			
Full IQ	95.8 ± 11.9	99.2 ± 10.1	.31
Verbal IQ	100.7 ± 12.3	98.1 ± 9.6	.45
Performance IQ	91.5 ± 13.5	100.6 ± 11.0	.02*
CY-BOCS total	22.4 ± 6.2		
CY-BOCS obsessions	11.2 ± 3.1		
CY-BOCS compulsions	11.2 ± 3.3		
CY-BOCS most severe	26.5 ± 6.1		
NIMH-OCS	9.3 ± 1.9	1.1 ± 0.4	<.01**
Handedness			
Right-handed	21 (95.5%)	21 (95.5%)	
Left-handed	1 (0.5%)	1 (0.5%)	
WSCT			
CA	4.1 ± 2.2	4.0 ± 1.9	.54
TE	16.7 ± 9.7	16.6 ± 9.9	.77
PE	3.8 ± 4.2	4.1 ± 5.7	.73

The demographic and clinical characteristics of the study subjects and performances on the WCST are shown.

*WCST*, Wisconsin Card Sorting Test; *OCD*, obsessive-compulsive disorder; *SD*, standard deviation; *IQ*, intelligence quotient; *CY-BOCS*, Children's Yale-Brown Obsessive Compulsive Scale; *CY-BOCS* most severe, CY-BOCS score during the period with the most severe symptoms; *NIMH-OCS*, National Institute of Mental Health Obsessive–Compulsive Scale; *CA*, categories achieved; *TE*, total errors; *PE*, perseverative errors.

* P < 0.05.

**P < 0.01.

**Figure 1:**
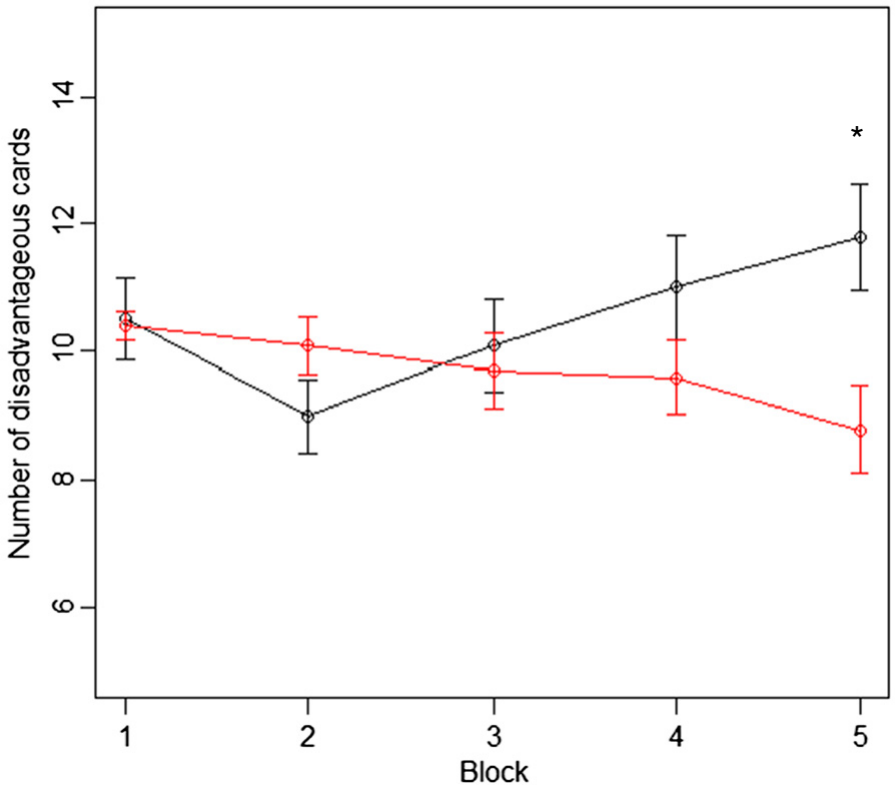
**Order of card selection from the 1st to the 100**^**th **^**trial.**The black and red lines show the mean number of disadvantageous cards drawn by OCD patients and controls, respectively, in each block of 20 cards. Horizontal bars indicate standard error of the means. Children with OCD chose more cards from the disadvantageous deck towards the end of the task. OCD, obsessive compulsive disorder; * Significantly higher compared to controls (Mann–Whitney test; P < 0.05).

In the last section of the IGT (selections 81–100), the number of disadvantageous card selections was significantly higher in the OCD group compared to the controls (shown in Table 2 and Figure 1). Regarding performance on the WCST, there were no significant differences in CA, TE, or PE between the OCD patients and the controls (shown in Table 1).

**Table 2:** The number of disadvantageous cards drawn in the Iowa Gambling Task

**Variable**	**OCD (n = 22)**	**Controls (n = 22)**	**P value**
	**Mean ± SD**	**Mean ± SD**	
No. of disadvantageous card selections (1–100)	52.3 ± 9.8	48.6 ± 7.9	.21
No. of disadvantageous card selections (1–20)	10.5 ± 3.0	10.4 ± 1.1	.50
No. of disadvantageous card selections (21–40)	9.0 ± 2.7	10.1 ± 2.1	.17
No. of disadvantageous card selections (41–60)	10.1 ± 3.4	9.7 ± 2.7	.68
No. of disadvantageous card selections (61–80)	11.0 ± 3.8	9.6 ± 2.7	.24
No. of disadvantageous card selections (81–100)	11.8 ± 3.9	8.8 ± 3.2	.03*

The mean number of disadvantageous cards drawn by OCD patients and controls in each block of 20 cards are shown.

OCD, obsessive-compulsive disorder; SD, standard deviation; No., Number.

* P < 0.05.

We also examined the associations between clinical parameters and the neuropsychological data. The number of disadvantageous cards selected in the last block of the IGT did not correlate significantly with age at onset, duration of illness, or CY-BOCS score at the time of assessment in the OCD group, or with current age, verbal IQ, or performance IQ in either the OCD or the control group (Pearson’s correlation; all p > 0.05). However, the CY-BOCS score during the period with the most severe symptoms in children with OCD significantly and positively correlated with the number of disadvantageous cards selected in the last block (r = 0.55, p < 0.01). The number of disadvantageous cards selected in the last block was also significantly greater in patients on antidepressant medications compared to patients not taking an antidepressant (Mann-Whitney’s test, p < 0.05). Total number of errors in the WCST was negatively correlated with current age in OCD patients (r = −0.44, p < 0.05) and with performance IQ in the controls (r = −0.55, p < 0.01); however, no other significant correlations were observed between WCST scores and the clinical parameters (all p > 0.05).

## Discussion

To the best of our knowledge, this is the first report on IGT performance in children and adolescents with OCD. The observed poor IGT performance suggests impaired decision-making function in OCD patients. Previous studies have described poor IGT performance in adult OCD patients [8-11] and one study demonstrated impaired IGT performance in patients with prominent hoarding symptoms [28], both of which are in line with our current findings regarding childhood-onset OCD. However, Nielen et al. [29] did not find significant differences in IGT performance between OCD patients and healthy volunteers.

Electrophysiological recordings of the OFC have revealed that orbitofrontal neurons play an important role in processing the motivational value of rewarding outcomes [30-32]. This fits very well with the fact that IGT performance is specifically sensitive to OFC activity. In the current study, children with OCD performed similar to controls in the early stages of the IGT, but selected more disadvantageous cards towards the end of the task. The persistent selection of cards from disadvantageous decks in IGT is a behavior also observed in patients with damage to the OFC [20][33]. Taken together, OFC dysfunction may be associated with the pathophysiology of childhood OCD.

The present findings that IGT performance correlated significantly with the CY-BOCS score during the period with the most severe symptoms but not with the CY-BOCS score at the time of the assessment suggest that IGT performance may be associated with the potential severity of OCD rather than the state of present symptom exacerbation. We infer that the poor performance on IGT in antidepressant users may be because antidepressants were more likely to be chosen as a treatment for those with severe symptoms during the initial clinical assessment. However, an alternative explanation may be that the negative effects of antidepressants on cognitive performance result in poor IGT performance. Further studies are needed to elucidate what factors influence IGT performance in children with OCD.

The current study showed no significant differences in performance on the WCST between OCD patients and controls. WCST performance is specifically related to function of the DLPFC [6], a region that has no direct influence on the orbitofrontal-striatal-thalamic circuit. Therefore, it is plausible that WCST performance is not as impaired as IGT performance in OCD.

The only previous study which examined WCST performance in children with OCD [34] reported reduced WCST scores in children with OCD compared to healthy controls, with effect sizes of d = 1.24 for total errors and d = 1.30 for categories completed. Our sample size had 97% power to detect an effect size of 1.2 at an alpha level of 0.05 (calculated by G*Power 3.1.3 [35]). Therefore, our results indicate that any potential difference in WCST performance between OCD patients and controls in the current study was smaller than that derived by Shin et al. [34]. We infer that the current inconsistency with the results of Shin et al. may be because patients and controls were not matched for IQ in their study.

There are numerous studies on WCST performance in adult OCD patients; however, results are inconsistent across studies. Several groups reported a reduced performance in OCD patients [9,36-38], while others reported no differences compared with healthy controls [39-42]. The inconsistencies across these studies may be attributable, in part, to the comorbidity of PDD or ADHD in some of the OCD patients. Children with high-functioning autistic disorder or ADHD display poor performance on the WCST [14]. To better understand and clarify these findings, any comorbidity with PDD or ADHD, the age of onset and duration of OCD, the intelligence of the subjects, and the age of assessment should all be taken into account.

There are several limitations to the current study. First of all, the number of participants was too small to draw definitive conclusions. Secondly, some children with OCD were on medication, which may have affected the results. Finally, we did not use neuroimaging methods such as fMRI to confirm our inference that the function of the OFC was associated with the results obtained in our study.

In summary, children with OCD displayed poor performance on IGT but did not differ on WCST scores compared to controls. These findings are in line with the hypothesis that OCD is associated with dysfunction of orbitofrontal-striatal-thalamic circuitry. However, large-scale studies combined with fMRI are needed to confirm the role of the orbitofrontal cortex in the pathophysiology of childhood OCD.

## Competing interests

The authors declare that they have no competing interests.

## Authors’ contributions

MaK designed the study and wrote the manuscript. MaK, YI, HU, MU and KW recruited and screened the study participants. MaK and YI diagnosed the study participants. MaK administered the IGT. MaK undertook the statistical analysis. AO and KS supervised the data analysis and writing of the paper. MoK, NS, and DS gave critical comments on the manuscript. All authors contributed to and have approved the final manuscript.
